# ZiBuPiYin recipe improves cognitive decline by regulating gut microbiota in Zucker diabetic fatty rats

**DOI:** 10.18632/oncotarget.14611

**Published:** 2017-01-12

**Authors:** Chunyan Gu, Wen Zhou, Wang Wang, Hong Xiang, Huiying Xu, Lina Liang, Hua Sui, Libin Zhan, Xiaoguang Lu

**Affiliations:** ^1^ School of Medicine and Life Science, Nanjing University of Chinese Medicine, Nanjing, Jiangsu, China; ^2^ Basic Medical College, Nanjing University of Chinese Medicine, Nanjing, Jiangsu, China; ^3^ The Second Affiliated Hospital of Dalian Medical University, Dalian, Liaoning, China; ^4^ Institute of Integrative Medicine, Dalian Medical University, Dalian, Liaoning, China; ^5^ Department of Emergency Medicine, Zhongshan Hospital, Dalian University, Dalian, Liaoning, China

**Keywords:** ZiBuPiYin recipe, diabetes, psychological-stress, cognitive decline, gut microbiota, Pathology Section

## Abstract

Numerous researches supported that microbiota can influence behavior and modulate cognitive function through “microbiota-gut-brain” axis. Our previous study has demonstrated that ZiBuPiYin recipe (ZBPYR) possesses excellent pharmacological effects against diabetes-associated cognitive decline. To elucidate the role of ZBPYR in regulating the balance of gut microbiota to improve psychological-stress-induced diabetes-associated cognitive decline (PSDACD), we compared blood glucose, behavioral and cognitive functions and diversity of the bacterial community among experimental groups. The Zucker diabetic fatty (ZDF) rats with PSDACD exhibited behavioral and cognitive anomalies showing as increased anxiety- and depression-like behaviors and decreased learning and memory abilities. High-throughput sequencing of the bacterial 16S rRNA gene revealed that *Roseburia* and *Coprococcus* were decreased in ZDF rats with PSDACD compared with control group. Notably, these changes were reversed by ZBPYR treatment. Our findings indicate that ZBPYR might prevent PSDACD by maintaining the compositions of gut microbiota, which could be developed as a new therapy for T2D with PSDACD.

## INTRODUCTION

Type 2 diabetes (T2D) is characterized by chronic hyperglycemia with progressive failure of pancreatic insulin secretion and increased peripheral insulin resistance [1]. More recent investigations show that cognitive impairment is found in T2D patients prior to the onset of these conditions [2–4]. Diabetes-associated cognitive decline (DACD), as slight cognitive decrements, is generally thought across species to a central nervous systems (CNS) complication of diabetes [5–7]. The development of DACD is a complex process involving genetic susceptibility and environmental factors [8, 9], yet the mechanisms have not been fully unmasked.

The immediate psychosocial stress (PS) is not thought to be the problem affecting health, however, chronic activation of PS is considered to be the key [10]. Chronic psychosocial stress increased the incidence of approaching impaired glucose tolerance to diabetes [11]. Previous reports deem the mechanism as that insulin sensitivity is declined and insulin resistance is enhanced under the control of the hypothalamus, the limbic system of the emotional loop [12, 13]. However, the exact mechanism of insulin signal transduction disorders inducing cognitive dysfunction is not well established. Since psychological-stress-induced diabetes-associated cognitive decline (PSDACD) adversely influences quality of life in T2D patients [13], developing a prospective strategy for PSDACD is of great importance.

ZiBuPiYin recipe (ZBPYR), a traditional formula of Chinese medicine documented in the book of Bujuji written by Wu Cheng in the Qing dynasty, is originated from Zicheng Decoction for clinical therapy of cognitive impairment [14]. Our previous work has demonstrated that ZBPYR improved DACD in db/db mice effectively [15], which examined the beneficial effects of ZBPYR on cognitive impairment and suggested that ZBPYR could increase brain leptin, improve insulin resistance and ameliorate peripheral high glucose environment. We also found that ZBPYR protected hippocampal neurons against Aβ amyloid- and glutamate-induced neurotoxicity [16, 17]. A recent study performed by our group revealed the characterization of gastrointestinal microbes (GM) in ZDF rat GI tract, suggesting that the microbiota might serve as a biomarker of impending or fully manifest T2D and altering the GI microbial communities might be a prospective strategy for treating obesity and T2D [18].

Hyperactive hypothalamus-pituitary-adrenal (HPA) axis leads to changes of the gut microbial interacting with the brain *via* the gut-brain axis to modulate brain development, stress responses, the overall glucose and lipid metabolism [19–22]. To elucidate the role of ZBPYR in regulating the balance of gut microbiota affecting psychological-stress-induced diabetes-associated cognitive decline (PSDACD), we used male Zuker diabetes fatty (ZDF) rat as T2D model, which presents developing hyperinsulinemia and hyperglycaemia [23] and is initiated with a mutation in the leptin receptor gene [24, 25].

## RESULTS

### ZBPYR improves glucose metabolism in ZDF rats exposed to PS

**Figure 1:**
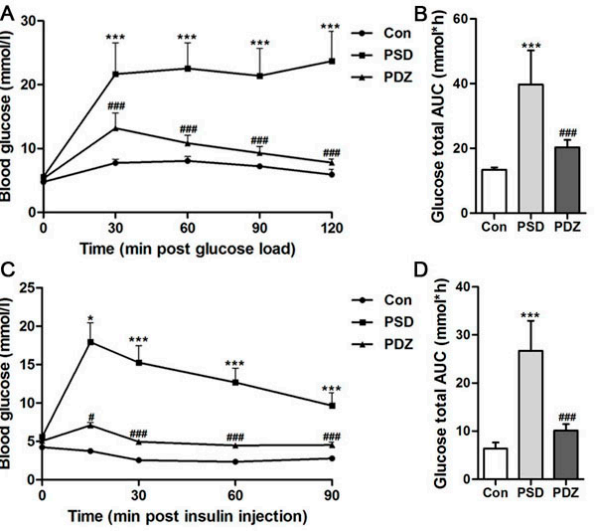
Effects of ZBPYR on glucose tolerance and insulin resistance in ZDF rats **A**. **B**. Oral glucose tolerance tests were examined in 14-hr-fasted rats after 10 weeks with high-fat diet. A: Blood glucose levels, **B**. Total glucose area under the curve (AUC). **C**. **D**. Insulin tolerance tests were conducted on 6-hr-fasted animals. **C**. Blood glucose levels, **D**. Total glucose AUC. Black circles, control (*n* = 3); black squares, PSD group (*n* = 3); black triangles, PDZ group (*n* = 3). *P < 0.05, ***P < 0.001 PSD *vs*. Con; #*P* < 0.05, ###*P* < 0.001 PDZ *vs*. PSD. Con: control ZDF rats; PSD: ZDF rats treated with PS; PDZ: ZDF rats treated with PS combining ZBPYR administration.

Three chronic PS (restriction, rotation, and congest) were imposed on ZDF rat to establish a model of PSDACD, while ZDF rat without PS was as control. As shown in Figure 1A, compared with controls, fasting blood glucose levels were remarkably elevated in PSD (ZDF rats with PSDACD) group at 30, 60, 90, and 120minutes (*P* < 0.01). The glucose total AUC of PSD group was extremely increased compared with controls (Figure 1B). Figure 1C and 1D showed that blood glucose concentrations in PSD rats were significantly higher than in control rats at 30, 60, 90 minutes (*P* < 0.05) and the glucose total AUC of PSD group was increased significantly compared with controls (*P* < 0.01). Administration of ZBPYR obviously reduced blood glucose levels and enhanced insulin sensitivity. Compared with PSD group, there were significant differences in PDZ (ZDF rats with PSDACD by ZBPYR treatment) group of the OGTT and ITT (*P* < 0.01), but the blood glucose did not recover to normal at the end point (Figure 1A-1D). The body weight of the rats in three groups was no significant differences (Table 1).

**Table 1:** Mean Body Weight (g) of rats in each group

Week	Con	PSD	PDZ
1W	144.33±4.89	148.48±3.63	145.14±8.42
2W	192.19±8.91	187.53±18.94	189.52±6.43
3W	236.48±11.60	214.93±22.04	226.87±12.35
4W	275.24±10.42	272.86±19.21	264.86±2.90
5W	304.86±9.50	298.38±17.35	291.71±1.03
6W	324.76±8.16	317.24±15.14	312.48±2.62
7W	340.76±9.70	329.62±11.55	334.29±3.71
8W	350.67±9.99	336.57±13.40	356.86±2.27
9W	354.00±13.00	339.33±17.36	366.86±5.58

### Scores on open field activity

**Figure 2:**
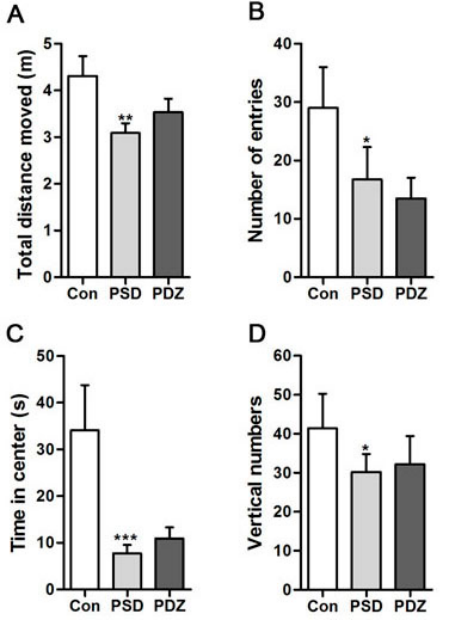
Parameters of activities in the open field test Total distances moved in the open field were measured. Vertical numbers during the experiment and numbers of entries into the center of the open field were counted. Time spent in the center of the open field was calculated. *P < 0.05, **P < 0.01, ***P < 0.001, PSD, PDZ *vs*. Con. Con: control ZDF rats; PSD: ZDF rats treated with PS; PDZ: ZDF rats treated with PS combining ZBPYR administration.

ZDF rats exposed to PS exhibited a series of abnormal behaviors, such as piloerection, indolent and fidget, which are typical anxiety- and depression-like behaviors. In the Open Field test, there were significant differences between the rats in Con group and PSD group, but no differences between Con group and PDZ group, indicating that the model of depressive-like behaviors was successfully established in ZDF rats. The PSD rats displayed being lack of physical activity (Figure 2A), less frequency entered into the center (Figure 2B), and shorter time in the center (Figure 2C) compared with controls. Vertical numbers of PSD rats were significantly decreased compared with controls (*P* < 0.05, Figure 2D). The behavior of rats in group PDZ resumed to normal levels after 8-week treatment of ZBPYR with the exposure to chronic restraint stress.

### ZBPYR improves spatial learning and memory performances

**Figure 3:**
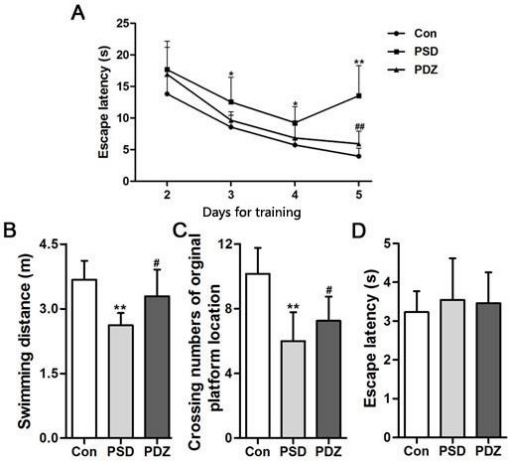
ZBPYR improves the performance of PSDACD rats in the Morris water maze test **A**. Learning performance of the animals was analyzed in the training trials by escape latency. PDZ rats presented shorter escape latency on the 4th and 5th day of training Black circles, control (*n* = 3); black squares, PSD group (*n* = 3); black triangles, PDZ (*n* = 3). **B**. **C**. Memory performance was investigated in the probe test. B: The swimming distances in training trials, **C**. The numbers of crossing over the original platform location. **D**. Escape latency was evaluated in the visible platform version of the Morris water maze. *P < 0.05, **P < 0.01 PSD *vs*. Con; #*P* < 0.05, ##*P* < 0.01 PDZ *vs*. PSD. Con: control ZDF rats; PSD: ZDF rats treated with PS; PDZ: ZDF rats treated with PS combining ZBPYR administration.

Morris water maze test was conducted in the last week (Week 16) to assess the spatial learning and memory performance of the rats. The escape latency of the control group was shorter than model groups from day 3 to 5 (*P* < 0.05), and progressively decreased over 4 days of training. The performances of the rats in PDZ group were close to control group and significantly strengthened compared with PSD group during days 3 to 5 (*P* < 0.05 at day 3, *P* < 0.01 at day 5) (Figure 3A). In the probe test, we observed that PDZ rats swam shorter distance to locate the original platform position than PSD rats (Figure 3B). A high frequency of crossing the target quadrant located with the platform was checked in PDZ group compared with PSD group (Figure 3C). There were no significant differences among the groups in terms of escape latency in the visible platform version (Figure 3D). These results indicated that ZBPYR administration evidently improved cognitive decline of ZDF rats with PSDCAD.

### ZBPYR regulates gut microbiota in ZDF rats with PSDCAD

**Figure 4:**
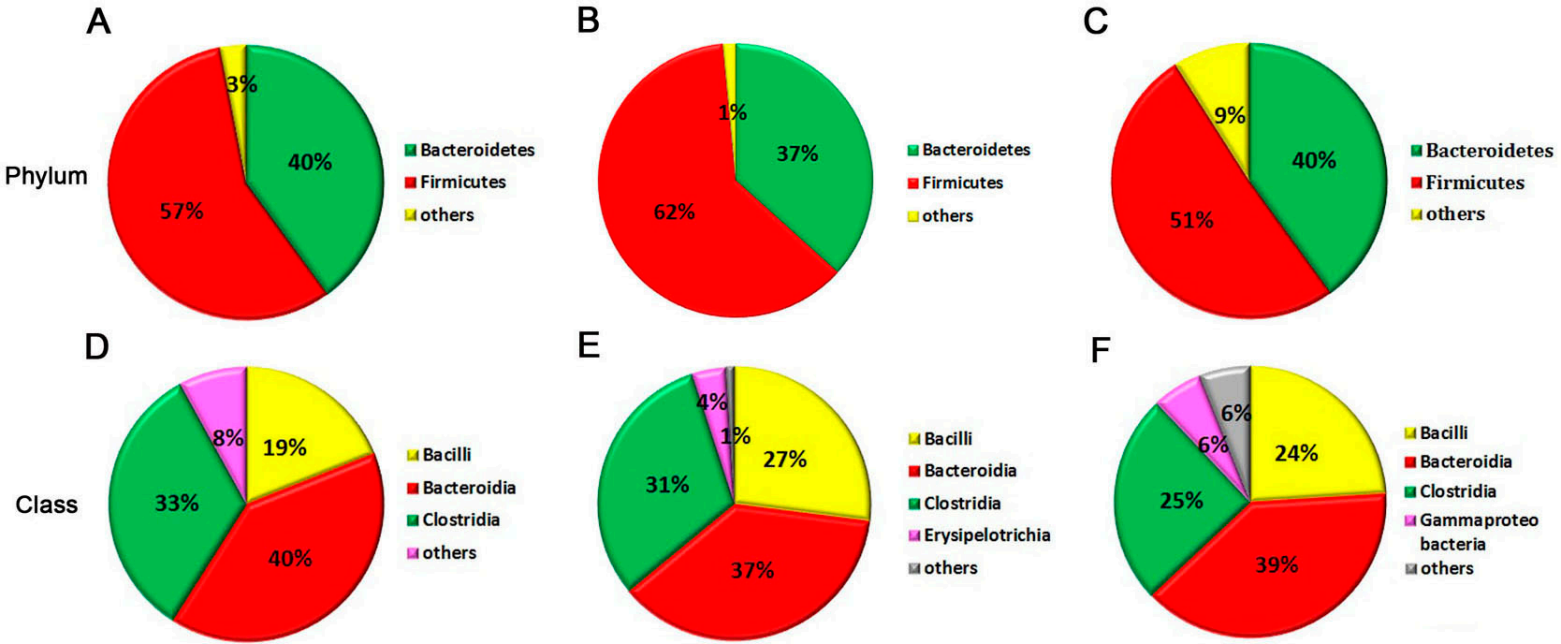
The “core” of intestinal community among Con, PSD and PDZ groups, respectively **A**-**C**: The distribution of intestinal community at the phyla level. **D**-**F**: The main bacterial class and the relative abundance of each bacteria. **A** and **D**: Control group; B and E: PSD group; C and F: PDZ group..

Gut microbiota provides a promising perspective to explore the pathological mechanism of PSDACD [26–29]. To reveal the link between T2D with PSDCAD and gut microbiota, the colons from three groups were sampled and sequenced. Microbial diversity analysis based on OTUs cluster suggested that the abundances of bacteria taxa even differ in the same anatomical sites. At phyla level, *Bacteroidetes* and *Firmicutes* were dominant bacteria among three groups (The total of their relative abundances was 97%, 99% and 91%, respectively), however, the slightly increased *Firmicutes* and decreased *Bacteroidetes* indicated that PSDACD may induce the varieties in the compositions of gut microbiota (Figure 4A-4C). The bacterial imbalance could be ameliorated by ZBPYR administration as the *Firmicutes/Bacteroidetes* ratio was decreased in PDZ group (Con, 1.49:1; PSD, 1.86:1; PDZ, 1.40:1). At class level, we identified a small ‘‘core’’ microbiota (mainly in the colon), including *Bacilli*, *Bacteroidia*, and *Clostridia*, and the relative percentages of these bacteria differ in the different groups shown in Figure 4D-4F.

**Figure 5:**
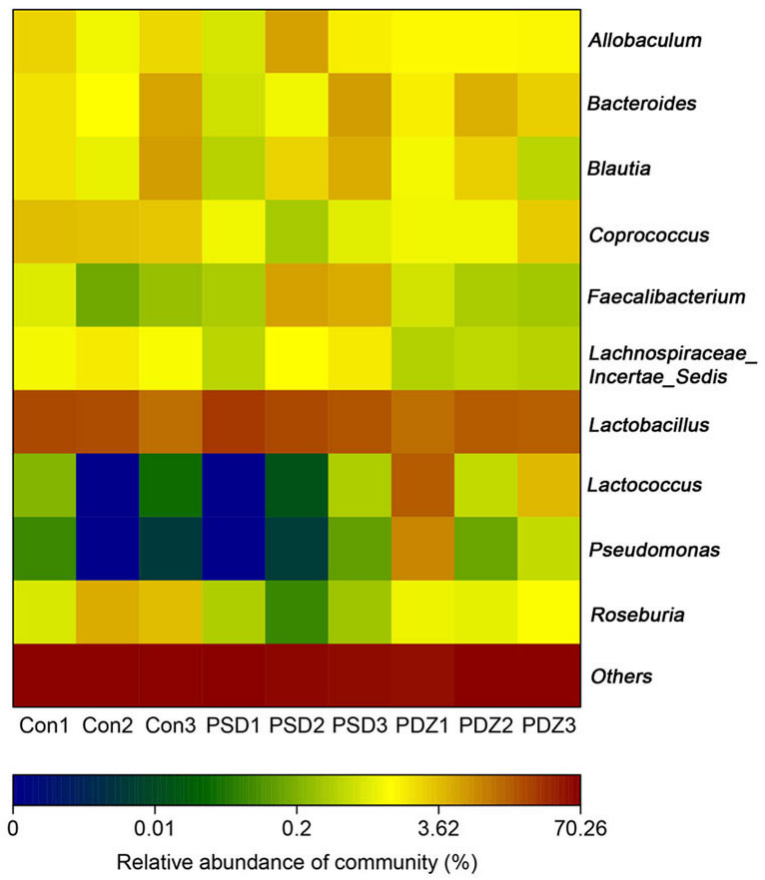
The heat map of 16S rRNA gene sequencing analysis of colonic mucosa at the genus level Heat map of the relative abundance of microbial species altered by PSDACD and ZBPYR treatment. Red colors indicate high values, whereas blue colors mean low values for the percent of reads classified at that rank.

**Figure 6:**
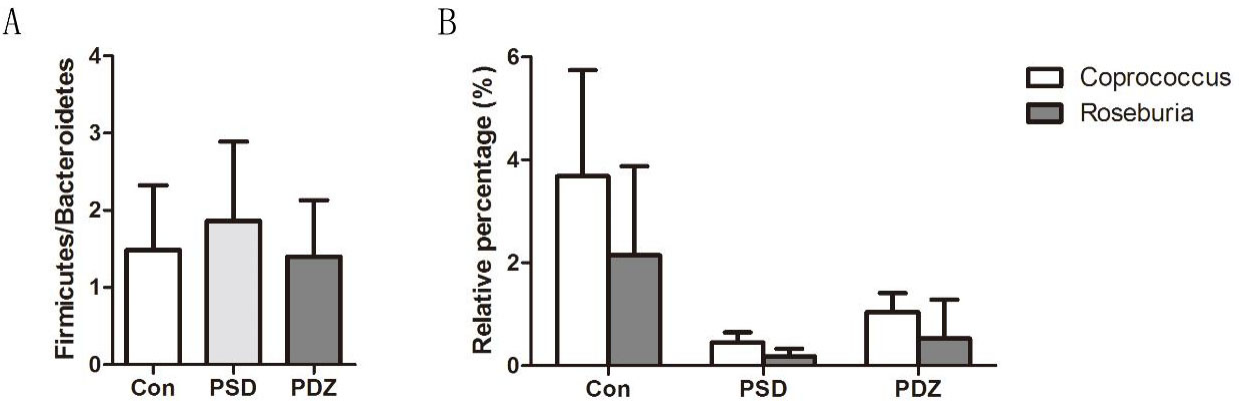
Impact of ZBPYR administration on the intestinal micro ecology balance **A**. *Firmicutes/Bacteroidetes* (F/B) ratios in three groups. **B**. 16S rRNA gene sequencing analysis of genus *Roseburia* and *Coprococcus* (*n* = 3, each group). Con: control ZDF rats; PSD: ZDF rats treated with PS; PDZ: ZDF rats treated with PS combining ZBPYR administration.

Heat map of the relative abundance of microbial species altered by PSDACD and ZBPYR treatment showed the differences of gut bacterial compositions compared among Con group, PSD group and PDZ group at the genus level (Figure 5). Consistent with previous reports, we found that the *Firmicutes/ Bacteroidetes* ratio was higher in PSD group compared with control group, in addition, it was recovered by ZBPYR administration in PDZ group (Figure 6A). The relative percentage of *Roseburia* was less in model group than that in control group (0.18% *vs*. 2.15%, Figure 6B), in contrast greater numbers of *Roseburia* were detected in PDZ group compared with PSD group (0.53% *vs*. 0.18%, Figure 6B). Interestingly, the analysis of *Coprococcus* followed similar trends as the relative percentage of *Roseburia*. These results confirmed that the homeostasis of the gut microbiota in PSDACD individuals was destroyed. ZBPYR could effectively manipulate the gut microbiota imbalance caused by PSDACD, mainly acting on *Roseburia* and *Coprococcus*.

## DISCUSSION

In recent years there is a growing interest in discovering correlations between gut microbiota composition/diversity and stress related disturbances [30]. Nishino et al. reported that the type of commensal microbiota located in the gut has an impact on behavior, such as anxiety-like behavior [31]. The reduction of total number of Xbp1pos lymphocytes in the gut closed to lymphoid tissue of the ileum in rats under induced chronic social stress, assuming the role of Xbp1 expression levels as a trigger of inflammation and potential link between stress and autoimmunity [32]. Recently, Naseribafrouei addressed that an overrepresentation of *Bacteroidetes* spp. and an underrepresentation of *Lachnospiraceae* were found in the fecal microbiota of 37 patients with depression [33].

It is well known that chronic psychological stress is associated with enhanced vulnerability to metabolic disturbance (obesity, insulin resistance and T2DM) through hypothalamic-pituitary-adrenal (HPA) axis hyperactivity [11, 34]. Over the past decade, an abundance of evidence indicated that diabetes and obesity are two metabolic diseases with insulin resistance and a low-grade inflammation [35–40]. Several studies showed that metabolic endotoxemia controls the inflammatory tone, obesity and diabetes, and high-fat diet alters gut microbiota and the plasma concentration of lipopolysaccharide (LPS) [41–43]. Despite these efforts attempted to clarify the correlation of microbiota, nervous system and chronic inflammatory diseases, the mechanism is still lacking.

Our previous study explored the effects of the ZBPYR on diabetes-related cognitive decline in db/db mice. ZBPYR improved learning and memory performance impairments, enhanced brain leptin, stimulating insulin signaling and prevented GSK3β overactivity, which disclosed that the benefit of ZBPYR to DACD may be by increasing dendritic spine density and reducing the injury factors of brain leptin and insulin signaling pathway [15]. In this study, Zucker diabetic fatty (ZDF) rat harboring a missense mutation (fatty, fa) in the leptin receptor gene (LEPR) [24] was used to determine whether ZBPYR improves cognitive decline by regulating gut microbiota. We found that ZBPYR reduced blood glucose concentration and promoted insulin sensitivity in ZDF rats with PSDACD (Figure 1A-D), and also enhanced learning and memory performance in PSDACD/ZBPYR rats (Figures 2-3). Recent research reported that the gut microbiota in T2DM patients is characterized by a decrease in the abundance of some universal butyrate-producing bacteria and greater number of opportunistic pathogens [44, 45]. Butyrate produced by microbial fermentation is a short-chain fatty acid, which can stimulate the pancreatic insulin secretion, increase insulin sensitivity and alter insulin signaling [46–48]. It exhibits remarkable functions, such as anti-obesity, alleviation of metabolic stress, protection from inflammatory response and improvement of cognition impairment [49, 50]. *Roseburia*, as a major butyrate producer, has a positive correlation with mental health [30]. And *Roseburia* could be increased by healthy diets to improve insulin sensitivity in obese population [51]. Moreover, decreased *Coprococcus* was found in obese patients with type 2 diabetes [52]. *Coprococcus* is significantly negatively correlated with LPS in plasma [53]. LPS can stimulate the secretion of proinflammatory cytokines, which ultimately impairs insulin sensitivity and promotes insulin resistance-related metabolic disorders [42]. Meanwhile, the abundance of *Coprococcus* was lower in mice exposed to social stressor compared to controls [54]. Interestingly, our findings pointed that PSDACD was correlated to the changes of gut microbiota characterized by reduced abundance of *Roseburia* and *Coprococcus* (Figure 6B), which were consistent with previous studies [44, 51–53, 55]. ZBPYR improved PSDCAD by increasing the relative abundance of beneficial bacteria, i.e. *Roseburia* and *Coprococcus* (Figure 6B).

In summary, our work provides evidences to support that the modulation of gut microbiota is associated with the development of PSDACD. We have successfully identified bacterial genus involved in the development of PSDACD, and defined intervention targets of ZBPYR in intestinal microecology for the first time. Inspiringly, these results highlight ZBPYR is a promising anti-PSDACD drug in adults with type 2 diabetes.

## METHODS AND MATERIALS

### Animals

Male 5-week-old obese Zuker diabetes fatty (ZDF) rats were purchased from Vital River Laboratories (VRL) (Beijing, China) and housed in the specific pathogen-free (SPF) animal experiment center at Dalian Medical University. The animals were fed a high-fat diet and water ad libitum and housed at 24°C ± 2°C with 65% ± 5% humidity with a 12 h light-dark cycle. All animal experiments were conducted in accordance with the National Institutes of Health Guide for the Care and Use of Laboratory Animals at Dalian Medical University (Dalian, China), which were approved by the Animal Ethics Committee of Dalian Medical University (Permit Number: SYXK (Liao) 2008-0002).

### Experimental protocol

After 1 week acclimatization, the obese ZDF rats were randomly distributed in 3 groups (n=3 each): ZDF control group, psychological-stress-induced Diabetes-Associated Cognitive Decline (PSD) group, and PS combined ZBPYR administration (PDZ) group. The PSD group and PDZ group were subjected to three stress stimulations: restriction, rotation, and congest. In the experiment of restricting stress, the animals were placed in the homemade rotating device rotating 15 min at 30 rpm with the interval ranging from 40-150 min. Four cycles were performed in each experiment every other day. During the rotation stress experiment, the rats were put in the opening bottles. The size of bottle is appropriate that the rat cannot be freely turned over. Whole experiment time was limited to 2 h, and was employed every other day. In the congest stress experiment, to analog crowded environment, 5 rats were kept in the same cage. It should be noted that rotation stress and restriction stress tests could not be carried out on the same day.

### Preparation and administration of ZBPYR

The ZBPYR consists of 12 herbs: Salvia miltiorrhiza Bge., Polygala tenuifolia Willd., Panax ginseng C.A.Mey., Dioscorea opposite Thunb., Poria cocos (Schw.) Wolf, Paronia lactiflora Pall., Dolichos lablab L., Acorus tatarinowii Schott, Santalum album L., Citrus reticulate Blanco., Nelumbo nucifera Gaertn. and Glycyrrhiza uralensis Fisch. All herbs were purchased from Dalian Lao Weixie outpatient department (Dalian, China). The mixtures were soaked in 8 volumes (v/w) of distilled water for 30 min and subsequently boiled for 90 min, then sandalwood was added. The decoction was filtered and concentrated before stored at 4°C. During a period of 8 weeks, ZBPYR was administered by oral gavage at a dose of 0.1 mL/10 g body weight in the treatment group, while PS and control group were orally administered an equal dose of ultrapure water (Milli-Q Integral Water Purification System, Millipore Corporation, Billerica, MA, USA).

### Oral glucose tolerance test and Insulin tolerance test

The control and ZDF rats were fasted for 14 h (overnight) followed by conducting an oral glucose tolerance test (OGTT) with a glucose solution in saline at 2 g/kg. Rats were fasted for 6 h before insulin tolerance test (ITT) and injected intraperitoneally with regular human insulin (0.75 U/kg body weight; Novolin, Novo Nordisk (China) Pharmaceutical Co., Ltd., Tianjin, China). Tail blood was sampled at 0, 30, 60 and 120 min after the glucose administration and at 0, 15, 30, 60, and 90 min after insulin injection, respectively. Blood glucose was then determined from tail blood using a glucometer (Roche, Mannheim, Germany).

### Behavioral experiments

Behavioral tests, including Open field tests and Morris water maze tests, were performed during the last 7 days of treatment according to the following schedule: Day 1 Open field test and Day 2–7 Morris water maze test. All procedures were conducted as previously described in detail [15, 56–58].

### Sample preparation

All rats were anesthetized with ether and decapitated. The colon samples were immediately dissected on ice, weighed and then rapidly frozen in liquid nitrogen. All samples were stored at −80°C.

### DNA Purification, 16S rRNA Gene Amplification and Illumina MiSeq sequencing

Genomic DNA was extracted from colon segments, the V1-V3 region of the bacteria 16S rRNA gene were amplified by PCR as previously described [18]. The barcode (an eight-base sequence) is unique to each sample. PCR reactions were performed as described previously [18]. Electrophoresis was used to isolate the PCR products on 2% agarose gels, and then the AxyPrep DNA Gel Extraction Kit (Axygen Biosciences, Union City, CA, U.S.) was applied to purify separated products followed by quantifying DNA using QuantiFluor™-ST (Promega, U.S.). The purified pooled products were sequenced on an Illumina MiSeq platform (Lingen Biotechnology Co., Ltd., Shanghai, China). The reads were denoised into the NCBI Sequence Read Archive (SRA) database, and sequences were further analyzed as previously described in detail [18, 49]. A total of 226054 high quality gene sequences were obtained from 9 rat colon samples with an average of 25117 sequences per sample (13736 to 30954 sequences).

### Statistical analyzes

All data were presented as means ± SD. Statistical analysis was evaluated by one-way ANOVA, followed by LSD (against all groups) post hoc with SPSS 17.0 (SPSS, Chicago, IL, USA). For data comparison, variables with non-Gaussian distribution were ASIN-square-root-transformed. P-values < 0.05 were considered statistically significant.
